# Pathological and prognostic role of mdig in pancreatic cancer

**DOI:** 10.18632/genesandcancer.149

**Published:** 2017-07

**Authors:** Srinivas Ashok Kumar, Chitra Thakur, Lingzhi Li, Hongjuan Cui, Fei Chen

**Affiliations:** ^1^ Department of Pharmaceutical Sciences, Eugene Applebaum College of Pharmacy and Health Sciences, Wayne State University, Detroit, MI, USA; ^2^ State Key Laboratory of Silkworm Genome Biology, The Institute of Sericulture and Systems Biology, Southwest University, Chongqing, China

**Keywords:** pancreatic cancer, mdig, alternative splicing, immunohistochemistry, survival rate

## Abstract

Pancreatic cancer is a highly aggressive malignant disease having very limited therapeutic options that ultimately results in its poor prognosis. It is still elusive on the etiology and tumorigenic mechanisms of pancreatic cancer. In the present report, we provide evidence showing involvement of the mineral dust-induced gene (mdig) in the pathogenesis and prognosis of the pancreatic cancer. Using immunohistochemistry approach on human pancreatic cancer tissue microarray, we found differential expression of mdig in pancreatic adenocarcinoma and normal pancreas. Based on the staining intensities of mdig in these tissue samples, we found that 12% of the cancer tissues were strongly positive for mdig, 39% and 31% were moderately and weakly positive respectively. Several alternatively spliced mdig mRNAs were detected in the selected pancreatic cancer cell lines. Through R2 platform for the patient survival analysis (http://r2.amc.nl), we found that enrichment of some specific exon of mdig predicates different survival rate of the pancreatic cancer patients. In summary, our findings may help in assessing the role of mdig in the pathogenesis of the pancreatic cancer and the prognosis of the pancreatic cancer patients.

## INTRODUCTION

Pancreatic cancer is the fourth leading cause of cancer death in both men and women in the United States [1]. In 2017, it is estimated that 53,670 Americans will be diagnosed and 43,090 patients will die of pancreatic cancer [2]. Most patients were diagnosed with pancreatic ductal adenocarcinoma. The 5-year survival rate after surgical resection is 20% and for those with metastatic disease, the 5-year survival rate is only about 2% [3]. Late detection and diagnosis has been the critical reason for poor survival rate in pancreatic cancer patients. About 85% of patients were in the advanced stages of the pancreatic cancer that is unresectable when diagnosed [1].

African Americans have appreciably higher rates of pancreatic cancer than whites. Heritable, germline mutations in p16, BRCA2 [4], and other genes appear to be associated with a total of 5%-10% of all pancreatic cancers, and penetrance of these mutations for the disease may be fairly low. Chronic pancreatitis of various types is associated with another 3%-4% of pancreatic cancer. Apart from germline mutations or pancreatitis, the other major risk factor associated with pancreatic cancer is cigarette smoking. Earlier studies suggested that patients with pancreatic cancer who smoked cigarettes were approximately 40% increased hazard for death compared with those who never smoked, and risk tends to increase according to frequency or duration of smoking [5]. Dietary studies have shown a fairly steady pattern of increased hazard with meat or cholesterol intake and decreased risk with fruit or vegetable consumption, although causal inferences regarding these associations are still uncertain [6].

The mineral dust- induced gene (mdig) was first identified in alveolar macrophages from patients with chronic lung diseases resulting from occupational exposure to mineral dust in mining industry [7]. This gene was independently characterized in human glioblastoma cell lines with an over expression of c-myc oncogene and named as myc-induced nuclear antigen 53 (MINA 53) [8] or nucleolar protein 52 (NO52) [9]. Mdig can be induced by a number of environmental hazards, such as arsenic, silica, coal dust, and particulate matter PM 2.5 [10]. The mdig gene encodes a 53 kDa protein that is involved in cell proliferation, neoplastic transformation, epigenetics, and immune regulation, and is over expressed in a variety of human neoplasms [10]. The mdig protein contains a conserved JmjC domain that is indicated in cell growth regulation, possibly through its effect on tri-methylation of lysine 9 on histone H3 (H3K9me3) and hydoxylase activity on ribosomal proteins [11, 12].

In the present study, we evaluated the expression of mdig in pancreatic adenocarcinoma cell lines at protein and mRNA levels. Using immunohistochemistry (IHC) approach, we detected expression of mdig in human pancreatic cancer tissues and assessed its association with clinicopathological features and prognosis. We showed that about 12% and 38% of total pancreatic malignant tissue were found to be strongly and moderately positive for mdig. We also demonstrated the presence of the alternatively spliced mdig mRNAs in pancreatic cancer cell lines we tested. Through R2: Genomics Analysis, we found opposite predictive power of different exon regions for the survival of the pancreatic cancer patients, which possibly suggested diversified roles of the alternatively spliced mdig mRNAs in the pathogenesis and prognosis of the pancreatic cancer.

## RESULTS

### Expression of mdig in human pancreatic adenocarcinoma cell lines

**Figure 1:**
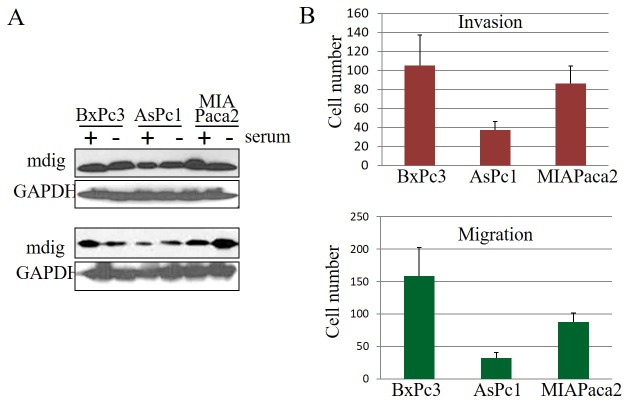
Expression of mdig in human pancreatic cancer cell lines **A.** The indicated cells were cultured in the medium with or without 5% of serum for 24 hours. Total cell lysates were used for Western blotting using antibodies against mdig and GAPDH. Two representative results were shown. **B.** Cell invasion and migration assays of the indicated three pancreatic cancer cell lines.

An over expression of mdig has been observed in a number of human cancers, implying its important role in the pathogenesis of human malignancies. To investigate whether mdig is over expressed in pancreatic cancer, the level of mdig expression was determined by western blot technique (Figure 1a). In three pancreatic cancer cell lines, Bxpc3, Aspc1 and MIAPaca2, we found that mdig protein was detected in these cell lines cultured in either presence or absence of serum. Several repeating experiments suggested that relative to the other two cell lines, the Aspc1 cells expressed lower level of mdig protein. Analysis of cell growth showed a similar proliferation rate among these three cell lines (data not shown). However, cell invasion and migration assays suggested that Bxpc3 cells have the highest, whereas Aspc1 cells have the lowest capabilities in migration and invasion (Figure 1b).

### Pancreatic adenocarcinoma cell lines express alternatively spliced mdig mRNAs

**Figure 2:**
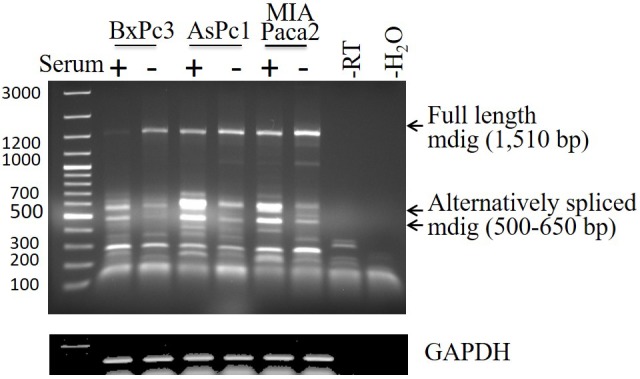
Expression and alternative splicing of mdig mRNA in pancreatic cancer cell line Total RNAs from the cells cultured in the conditions as described in Figure 1. RT-PCR was performed using the primer set that amplifies full length mdig mRNA with a size of 1,510bp. The PCR products were separated on 1% agarose gel. Arrows indicates full length and the alternatively spliced mdig mRNAs.

In an effort to determine the mRNA levels of mdig among these pancreatic cancer cell lines, a traditional RT-PCR was applied for the detection of mdig mRNAs. The PCR primer set was derived from exon 1 and exon 10 regions of mdig gene, respectively, which amplifies a 1,510 bp fragment of mdig mRNA encompassing the entire open reading frame. A marginal increased expression of the constitutive full length mdig mRNA was noted among these cell lines cultured in the serum-free medium (Figure 2). We had previously reported that there are alternatively spliced mdig mRNAs in which the entire exon 2 region was spliced out with or without inclusion of an alternative exon 5’ in lung epithelial cells and lung cancer cells [7]. Such alternatively spliced mdig mRNAs with sizes around 500 to 650 bp were detected in all of these pancreatic cancer cell lines (Figure 2). In contrast to the constitutive full length 1,510 bp mdig mRNA, the expression level of the alternatively spliced mdig mRNAs was decreased in the cells cultured in serum-free medium, suggesting different regulatory mechanisms for the expression of the constitutive full length and alternatively spliced mdig mRNAs.

### Correlation of mdig expression and the pathogenesis of pancreatic cancer

**Figure 3:**
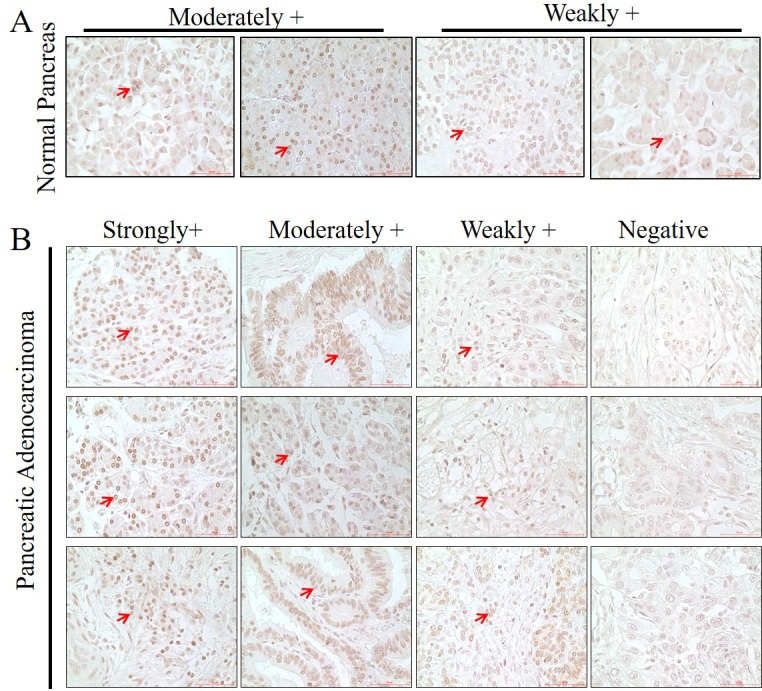
Immunohistochemical analysis of mdig protein in the human pancreatic tissue and pancreatic adenocarcinoma tissue microarray Paraffin embedded tissue microarray slide was immunostained for mdig protein as described in Materials and Methods. Red arrows depict the cells positive for mdig. Scale = 50 μM; Magnification = 40X. A. Normal pancreatic tissues; B. Pancreatic adenocarcinoma.

We and others had demonstrated an association of mdig expression and the pathogenesis of lung cancer, breast cancer and other cancers. To determine whether there is a correlation between mdig expression and the clinical characteristics of the human pancreatic cancer, we measured the protein level of mdig among the pancreatic tissues through immunohistochemistry on a tissue microarray containing 42 cases of pancreatic cancer tissues and 6 normal pancreatic tissues. Based on the staining intensities of mdig, we found that in the normal pancreatic tissues 34% was moderately positive and 66% was weakly positive, no strongly positive staining of mdig was detected (Figure 3a). However, in the pancreatic malignant tissue, we found 12% strong positive, 39% moderately positive, 31% weakly positive and 18% negative (Figure 3b).

**Figure 4:**
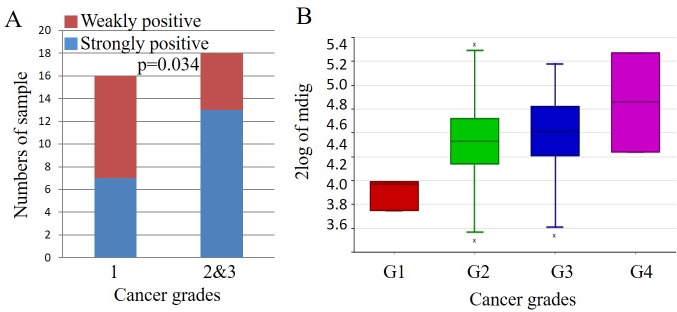
Association of mdig expression with the grades of pancreatic adenocarcinomas **A.** Quantification of relative mdig expression in tissue microarray containing 42 cases of pancreatic adenocarcinoma tissue samples at different stages. **B.** Mdig expression profiles among 130 cases of pancreatic cancer at grades 1, 2, 3, and 4 as deposited in the R2: genomics database (https://hgserver1.amc.nl/cgi-bin/r2/main.cgi).

To further evaluate mdig expression and the pathogenesis of pancreatic cancer tissues, we determined the protein levels of mdig by IHC analysis among the pancreatic cancer tissues with well-defined clinicopathological features. The association between mdig expression and clinicopathological variables in pancreatic cancer patients was analyzed additionally by chi-square test (Table 1). Among these variables, we found that cancer grades 2 and 3 exhibited a significantly strong mdig expression than the pancreatic cancer tissues at grade 1 (*p* = 0.03) (Figure 4a). No other clinicopathological variables showed a significant correlation with mdig expression. This observation is in agreement with mdig expression profiles among 130 cases containing grades 1, 2, 3, and 4 pancreatic cancer (Figure 4b), in which grade 1 showed the lowest, and grade 4 showed the highest level of mdig expression.

**Table 1:** Expression levels of mdig and the clinicopathological variables of the pancreatic cancer patients Pearson chi-square tests was performed to determine the *p*-value.

Variables	No.	Mdig expression		*P*-value
		Low No. (%)	High No. (%)	
Gender				0.695
Male	12	6	6	
Female	30	13	17	
Age (years)				0.118
> 60	30	12	18	
< 60	12	8	4	
Cancer grade*				**0.034**
1	19	12	7	
2 & 3	23	7	16	

### Correlation between exon specific mdig expression and the overall survival of pancreatic cancer patients

**Figure 5:**
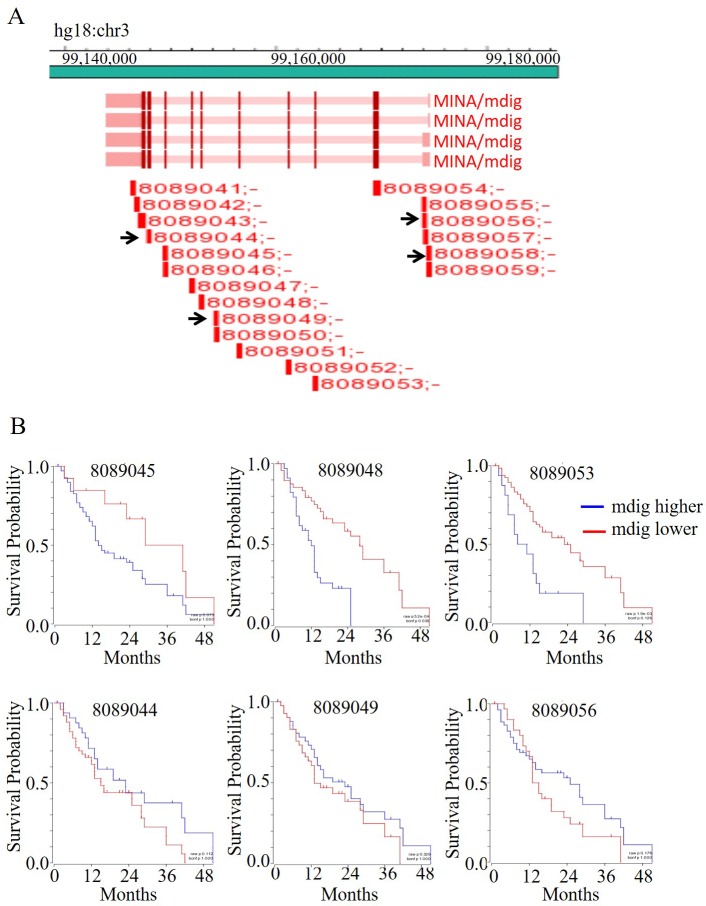
The levels of mdig expression predict survival of the patients with pancreatic cancer **A.** Diagram of mdig gene structure and the positions of the probe detected. **B.** Kaplan-Meier survival analysis of the selected probe sets as indicated in the R2: Genomics Analysis and Visualization Platform database. Y axis depicts survival probability and X axis depicts months.

Our earlier studies suggested that increased expression of mdig predicts poorer overall survival of breast cancer and lung cancer patients. Studies by other groups also indicated poorer survival of the hepatocellular carcinoma with higher level of mdig [13]. To reveal the potential prognostic value of mdig expression for the pancreatic cancer patients, we used R2 genomics visualization tool in a Mixed Tumor Pancreas (Exon) dataset to investigate the changes in the patient survival rate with respect to individual mdig exon that was detected by 19 probes provided by Affymetrix (Figure 5a). Kaplan-Meier survival graphs were generated from this data set and were compared based on their survival outcomes. Among these 19 survival analysis based on the individual probes for different exons, 15 probe sets indicated a poorer survival of the patients with higher mdig expression. However, 4 probes, probe sets 8089044, 8089049, 8089056, and 8089058, which correspond to exon 2, alternative exon 5, and exon 10, predicted better survival of the patients with higher mdig expression (Figure 5b). Since missing exon 2 and inclusion of an alternative exon 5 had been found in an alternatively spliced mdig mRNA we identified previously, the survival profiles of the different mdig exons suggested a unique or opposite role of the alternatively spliced mdig relative to the constitutive full length mdig in the pathogenesis of the pancreatic cancer.

## DISCUSSION

There has been a decline in the death rate for the cancers in lung, colorectal, breast, and prostate, since 2003. In contrast, the death rate of pancreatic cancer was increasing in the same time period. The five year survival rate of pancreatic cancer is only about 6% since 1970s [14]. We and others had shown an increased expression of mdig in a number of human cancers, including lung cancer, colon cancer, and breast cancer, which implies important contribution of mdig towards the pathogenesis of human cancers [11]. However, there are no reports studying the relationship of mdig expression and the clinicopathological features and/or prognosis of the pancreatic cancer. In the current study, we provide evidence showing that high level of mdig expression was a salient feature in human pancreatic cancer cell lines and tissue. By western blot analysis, we observed that all three pancreatic cancer cell lines, Bxpc3, Aspc1 and MIAPaca2, expressed high level of mdig protein, although there was a slight difference in the expression levels of mdig among these cell lines studied. Bxpc3 and MIAPaca2 showed high mdig expression levels compared to Aspc1. The RT-PCR analysis showed mdig mRNA expression in all three cell lines. Interestingly, we were able to detect some strong bands of mdig mRNAs resulted from alternative splicing. The alternatively spliced mdig mRNAs might have distinctive prognostic values against the normally spliced mdig mRNA on the overall survival of the pancreatic cancer patients.

Despite extensive studies, the etiology and mechanisms of the pancreatic cancer are still elusive. Some epidemiologic studies suggested a higher incidence rate of pancreatic cancer among African Americans than Whites. Several heritable, germline mutations in p16 and/or BRCA2 may be associated with the development of pancreatic cancer [4]. It was estimated that 95% of pancreatic carcinoma showed inactivation of p16 tumor suppressor [15]. P16 is located on chromosome 9, where it encodes a protein that inhibits entry into the S phase of the cell cycle by inhibiting cyclin-dependent kinase (CDK) 4/6-dependent phosphorylation of retinoblastoma (RB) protein. Inactivation of p16 leads to an uncontrolled cell growth due to a fast cell cycle transition [16]. Intragenic mutation, loss of heterozygosity (LOH), homozygous deletion, and promoter hypermethylation, may be responsible for p16 inactivation. In addition to p16 inactivation, about 50% to 75% of pancreatic cancer show dysfunction of p53, one of the most important tumor suppressors [17]. As a transcription factor whose gene is located on chromosome 17, p53 regulates a variety of signaling pathways linked to cell cycle arrest, apoptosis, and DNA repair. Accordingly, functional inhibition of p53 will lead to loss of cell cycle “check-point” [16, 18].

Mutations in K-ras gene were frequently observed in a number of human cancers. This mutation was also found in almost all resected cases of pancreatic cancer [19]. It was believed that K-ras mutation occurs in the early stage of cancer development [20]. Because of the proximity of K-ras gene with other ras family of GTP-binding proteins on chromosome 12, genetic abnormality of K-ras gene may be additionally associated with the functional disruption of other GTP-binding proteins critical for cell growth, differentiation and survival.

Chronic pancreatitis of various types was considered as a contributing factor for about 3%-4% of pancreatic cancer. Apart from germline mutations or pancreatitis, other major risk factors associated with pancreatic cancer include cigarette smoking, obesity, diabetes, cirrhosis of the liver, etc. Earlier studies suggested an approximately 40% increased hazard for death of the pancreatic cancer patients who smoked cigarettes compared with those who never smoked, and risk tends to increase according to frequency or duration of smoking [5]. Dietary studies have shown a fairly steady pattern of increased hazard with meat or cholesterol intake and decreased risk with fruit or vegetable consumption, although causal inferences regarding these associations are still uncertain [6].

We had originally identified alternatively spliced mdig mRNAs in human cancer cell lines [7]. One of these alternatively spliced mdig mRNAs lacks the entire exon 2 region that encodes the N-terminal and the partial JmjC regions of the mdig protein. This alternatively spliced mdig mRNA was additionally identified recently in the transformed cells induced by environmental carcinogen arsenic (Li, et al, unpublished observation). The JmjC domain of mdig was considered as the potential region for protein hydroxylation and/or demethylation. Loss of JmjC domain due to alternative splicing of the mdig mRNA, thus, may impair the function of mdig protein or endue new function of this protein. In pancreatic cancer cell line, such alternatively spliced mdig mRNA were also identified (Figure 2). Although it remains to be determined on the functional aspects of the alternatively spliced mdig mRNAs, the opposite prediction of the pancreatic cancer patients by the probe sets against different exons of the mdig mRNA suggests unique functions of the alternatively spliced mdig mRNAs. We are currently trying to distinguish these functional differences between the constitutively spliced full-length mdig and the alternatively spliced mdig mRNAs in both normal and cancer tissues.

In the current report, we provided evidence showing association of mdig expression and the pathogenesis of the pancreatic cancer. Previous studies suggested that increased expression of mdig predicts poor survival of the patients with breast cancer, lung cancer and hepatocellular carcinoma [12, 13, 21]. By using R2 genomics visualization tool, the probe-exon-based analysis also suggested an inverse relationship between the levels of mdig expression and the survival of the pancreatic cancer patients. However, 4 out of 19 probes showed that increased mdig expression, in fact, predicts a better overall survival of the pancreatic cancer patients, which most likely indicated a different or opposite function of the alternatively spliced mdig mRNAs. It remains to be further investigated on how different isoforms of mdig resulted from the normal and alternative splicing affect the initiation, progression and pathogenesis of the pancreatic cancer. It will also be important to determine whether mdig can serve as a biomarker for diagnosis, prognosis and molecular targeting therapy for pancreatic cancer.

## MATERIALS AND METHODS

### Cell culture

The human pancreatic adenocarcinoma (PA) cell line Bxpc3, Aspc1 and MIAPaca2 were purchased from the American Type Culture Collection (ATCC, Manassas, VA). Aspc1 and MIAPaca2 cells were cultured in Dulbecco's Modified Eagle's Medium - high glucose (DMEM) with 10% fetal bovine serum (Invitrogen), 1% penicillin-streptomycin. Bxpc3 cells were cultured in RPMI-1640 medium (HyClone) with 10% fetal bovine serum and 1% penicillin-streptomycin and in presence of 5% CO_2_.

### Western blotting

Total cellular proteins were prepared by lysing the cells in RIPA buffer (Millipore, Billerica, MA) supplemented with phosphatase/protease inhibitor cocktail and 1 mM PMSF through sonication and centrifugation, followed by quantification using a Micro BCA Protein Assay Reagent Kit (Thermo Scientific, Pittsburgh, PA). Before loading on 10% SDS-PAGE gels, the proteins were boiled in LDS sample buffer (Invitrogen) containing 1 mM dithiothreitol. The separated proteins were transferred onto PVDF membranes (Invitrogen). Membranes were probed with the primary antibody at a dilution of 1:1000 or 1:2000 (according to signal intensity) overnight at 4 °C. The secondary antibody with HRP was applied at the dilution of 1:2000. The mdig antibody was purchased from Invitrogen and GAPDH was purchased from Cell Signaling Technology, Inc.

### RT-PCR

Total RNA was prepared by lysing the cells with TRIzol reagent (Invitrogen). Access Quick RT-PCR System (Promega) was used for reverse transcription and PCR. Sample composed of 1 μg of total RNA and 0.3 μM sense and anti-sense primer. The mdig primer sequences are: left primer: 5′-TCATGTCGGGCCTAAGAGAC-3′; right primer: 5′-GGCATTTGATTCTGCAAAGG-3′, which amplify a 1509 bp cDNA fragment covering the entire coding region of the mdig mRNA. The GAPDH primer sequences are: sense: 5′-CTGAACGGGAAGCTCACTGGCATGGCCT-3′; antisense: 5′CATGAGGTCCACCACCCTGTTGCTGTAG-3′.

### Immunohistochemistry

The tissue microarray slide BC14012 was purchased from US Biomax, Inc. (Rockville, MD) and deparaffinized with xylene and hydrated in series of alcohol. Slides were incubated with 1.5% H_2_O_2_ for 20 min at room temperature, to quench the endogenous peroxidase activity. Non-specific binding of immunoglobulin was blocked by incubating slides in a solution consisting of 5% goat serum and 0.2% triton-X 100 in PBS for 2 h at room temperature. Proceeded by incubation with monoclonal antibody against Mdig/Mina 53 (mouse anti-Mina 53, Invitrogen) overnight at 4°C in 1:100 dilutions. Next day, goat anti-mouse biotinylated secondary antibody was applied at 1:200 dilution and incubated for 2 h at room temperature. Slides were then incubated with an ABC reagent (Vectastain Elite ABC kit) for 45 min at room temperature and the chromogen was developed with diaminobenzidine (DAB). Hematoxylin was used as counterstain and it was mounted with entellan. All incubation steps were carried out in a humidified chamber and all washing steps were performed with 1 × PBS. Bright field optics of the Nikon Eclipse Ti-S Inverted microscope (Mager Scientific, Dexter MI, USA) was used to capture the images.

### Migration and invasion

Cell migration and invasion were determined using BD BioCoat^TM^ Matrigel^TM^ Invasion and Migration Chambers according to the manufacturer's instruction. After incubating for 24h, the cells in the upper chambers were scrubbed out using cotton tipped swab. The cells on the lower surface of the membrane were stained with the Diff-Quik Kit. The migrated and invasive cells were counted under a bright field microscope.

### Statistics

The measurement errors for quantitative experiments were determined using standard deviation (SD). Comparison between mdig expression and clinicopathologic variables were determined by chi-square test. Differences with a *P* value of ≤ 0.05 were considered significant. Data were analyzed using IBM SPSS Statistics 22.0 (SPSS, Chicago, IL, USA). R2: Genomics Analysis and Visualization Platform (http://r2.amc.nl) a public data set was used for analyses and generation of Kaplan-Meier survival curve.
